# Achieving Accurate Automatic Sleep Staging on Manually Pre-processed EEG Data Through Synchronization Feature Extraction and Graph Metrics

**DOI:** 10.3389/fnhum.2018.00110

**Published:** 2018-03-23

**Authors:** Panteleimon Chriskos, Christos A. Frantzidis, Polyxeni T. Gkivogkli, Panagiotis D. Bamidis, Chrysoula Kourtidou-Papadeli

**Affiliations:** ^1^Laboratory of Medical Physics, Medical School, Aristotle University of Thessaloniki, Thessaloniki, Greece; ^2^Greek Aerospace Medical Association and Space Research, Thessaloniki, Greece; ^3^Director Aeromedical Center of Thessaloniki, Thessaloniki, Greece

**Keywords:** computerized sleep staging, electroencephalogram, feature extraction, functional connectivity, graph theory, classification, artificial intelligence

## Abstract

Sleep staging, the process of assigning labels to epochs of sleep, depending on the stage of sleep they belong, is an arduous, time consuming and error prone process as the initial recordings are quite often polluted by noise from different sources. To properly analyze such data and extract clinical knowledge, noise components must be removed or alleviated. In this paper a pre-processing and subsequent sleep staging pipeline for the sleep analysis of electroencephalographic signals is described. Two novel methods of functional connectivity estimation (Synchronization Likelihood/SL and Relative Wavelet Entropy/RWE) are comparatively investigated for automatic sleep staging through manually pre-processed electroencephalographic recordings. A multi-step process that renders signals suitable for further analysis is initially described. Then, two methods that rely on extracting synchronization features from electroencephalographic recordings to achieve computerized sleep staging are proposed, based on bivariate features which provide a functional overview of the brain network, contrary to most proposed methods that rely on extracting univariate time and frequency features. Annotation of sleep epochs is achieved through the presented feature extraction methods by training classifiers, which are in turn able to accurately classify new epochs. Analysis of data from sleep experiments on a randomized, controlled bed-rest study, which was organized by the European Space Agency and was conducted in the “ENVIHAB” facility of the Institute of Aerospace Medicine at the German Aerospace Center (DLR) in Cologne, Germany attains high accuracy rates, over 90% based on ground truth that resulted from manual sleep staging by two experienced sleep experts. Therefore, it can be concluded that the above feature extraction methods are suitable for semi-automatic sleep staging.

## Introduction

Sleep is a biological process that is essential for human physical and mental health. Sleep quality is associated with quality of life (Reid et al., 2006; Ioannides et al., 2017) and, therefore, studying sleep mechanisms may provide insights into various diseases such as diabetes, cardiovascular diseases and memory impairment (Skeldon et al., 2014). Sleep quality may be evaluated in terms of duration as well as the duration of each sleep stage (Buysse et al., 1989). According to the American Sleep Medical Association, sleep is initially divided in two main types: Rapid Eye Movement sleep (REM) and Non Rapid Eye Movement sleep NREM (AASM, 2007). Normal sleep proceeds in 90-min cycles of REM and NREM stages (Rechtschaffen and Kales, 1968). The latter type is further divided in three stages namely N1, N2 and N3. This categorization results in a total of four sleep stages, and is based on the different electroencephalographic (EEG) rhythms that are observed during each of the sleep stages.

Assigning sleep stages to a given portion of an electroencephalographic recording is known as sleep staging and is usually conducted by specialized experts (Agarwal and Gotman, 2001). This process, however, is cumbersome, error prone and time consuming and delays further data processing (Younes, 2017). Assuming normal sleep duration equal to 8 h, during which the EEG is recorded, these recordings are split into 30 s epochs, resulting in about 960 epochs that must be assigned to a sleep stage. When multiple recordings are gathered during a study that involves a group of participants, the task of sleep staging appears daunting. As a result many methods have been proposed for automatic sleep staging in order to reduce the time required, effort spent and number of errors (Younes, 2017).

In most automatic sleep staging methods proposed, sleep stage classification is achieved by combining time and frequency linear and non-linear features calculated from EEG signals; with maximum accuracy rates reaching from 80% (Radha et al., 2014) to 94% (Lajnef et al., 2015). In (Bajaj and Pachori, 2013) authors use the time-frequency image of EEG signals to achieve automatic sleep staging which is calculated through the smoothed pseudo Wigner–Ville distribution, achieving close to 93% accuracy. In Hassan and Bhuiyan (2016a) and Hassan and Bhuiyan (2016b) an estimation of the initial signal in the time-frequency domain is achieved through Empirical Mode Decomposition reporting very high accuracy rates in some cases exceeding 95%. Other approaches in sleep stage classification include energy features achieving about maximum 87% accuracy (Hsu et al., 2013) or entropy features reporting an accuracy of around 80% (Rodríguez-Sotelo et al., 2014). Despite these high accuracy rates, there is lack of information regarding the functional co-operation among electrode sites. This type of information is hypothesized to produce novel insights into the brain macro-architecture during sleep (Ioannides et al., 2017) and to enhance the algorithm's applicability into generic sleep datasets other than the employed training and validation sets.

More specifically, most of the aforementioned approaches focus on specific EEG properties either on time-frequency features (Bajaj and Pachori, 2013; Radha et al., 2014; Lajnef et al., 2015; Hassan and Bhuiyan, 2016a,b) or energy and entropy features (Hsu et al., 2013; Rodríguez-Sotelo et al., 2014). These properties track the main sleep characteristics on single channels and the EEG grapho-elements (K-complexes and spindles) that were used to perform automatic sleep staging. However, they do not consider the sensor level activity among distant electrode locations during sleep. Coupling among electrodes and more importantly among brain regions have demonstrated to play a pivotal role to the consciousness level (Horovitz et al., 2009). Previous studies have demonstrated that the sleep deepening is associated with disconnection of key nodes of the Default Mode Network (DMN) even from the early sleep stages (Sämann et al., 2011). These neuroscience findings highlight the significance of a new approach for semi-automatic sleep staging which regards the brain activity as an integrated functional network with dynamic co-operative activity among its key elements (nodes).

Aiming to enhance the field of computerized sleep staging based on macroscopic investigation of the brain function (system level), the approach presented in this paper employs contemporary mathematical tools quantifying the EEG functional connectivity during sleep through the notions of the Synchronization Likelihood/SL (Stam and Van Dijk, 2002) and Relative Wavelet Entropy/RWE (Rosso et al., 2001). Synchronization metrics have been used to study brain connectivity on various aspects of sleep. To begin with, it has been shown that sleep deprivation impacts functional connectivity and graph metrics of prefrontal cortical areas (Verweij et al., 2010) leading to structural changes in these networks which affect cognitive performance. In (Vecchio et al., 2017) the authors studied cortical connectivity during sleep onset and conclude that the brain network is less ordered in the sigma frequency band and displays higher order in lower frequency bands. Synchronization has also been used to study the dynamics within sleep stages, as is the case in Achermann et al. (2016) where the authors conclude that REM sleep is the most synchronized brain state in humans. Furthermore, the stability of brain connectivity was studied in Jobst et al. (2017) through brain computational modeling. Despite the use of synchronization in other sleep aspects, we found no prior work which utilizes synchronization metrics for automatic sleep staging.

In our work, after the calculation of synchronization metrics, electrode network analysis was conducted through graph theory, during which global functional characteristics were calculated namely the small-world property, cluster coefficient, characteristic path length, node degree and network density (Watts and Strogatz, 1998; Nicosia et al., 2013; Frantzidis et al., 2014b). Both the synchronization and the graph metrics mentioned above are expected to fluctuate during the course of sleep and these fluctuations are hypothesized to be more prominent between the different stages of sleep. It must be noted that minor differences arise during the calculation of the above metrics since the synchronization matrix given through synchronization likelihood is undirected, while the one given through relative wavelet entropy is directed. For instance the values of graph density and node degrees are omitted from the wavelet entropy graphs as they contain the same values over all epochs, due to the fact that these graphs are both directed and fully connected. This fact introduces similarities between sleep epochs belonging to different sleep stages which are undesirable as it will render differentiation between the various sleep stages harder for the classifiers. In both cases mentioned above the calculation and use of graph metrics is bound to further quantify and describe the synchronization and cooperation of the various brain regions on a higher level than that provided by the synchronization values, rendering the sleep stage classification task easier. The aim of this study is to investigate whether a system level notion of computerized sleep stage is feasible by presenting early results deviating from the traditional time and frequency analysis. It ventures into calculating features that describe the functional interactions between electrode sites and how these vary during the different sleep stages. Furthermore the accuracy rates achieved through the experimental procedures are comparable to other state-of-the-art methods.

## Materials and methods

The pre-processing pipeline described in section Pre-processing pipeline was applied on the entire polysomnographic (PSG) data derived from each participant and divided into five (5) segments. sections Visual Sleep Scoring and Synchronization feature extraction describe respectively the visual sleep scoring and the feature extraction procedures on epoch-level. Finally, section Classification Methodology Component describes the classification methodology which was applied on the group level (features from all the participants).

### Experimental set-up and initial data

The full experimental set-up is described in (Kramer et al., 2017) and was performed in the “ENVIHAB” premises of the German Aerospace Agency (DLR). The study was funded by the European Space Agency (ESA) following a 60-day long bed rest head down protocol aiming to simulate a microgravity environment on 23 healthy male adults between the ages of 23 and 45 (mean: 29 ± 6 years). The participants signed a written informed consent for the various parts of the study, as described in (Kramer et al., 2017). The study was based on specific guidelines and regulations, which were approved by the ethics committee of the Northern Rhine Medical Association (Arztekammer Nordrhein) in Duesseldorf, Germany, and the Federal Office for Radiation Protection (Bundesamt für Strahlenschutz). The aim of the experiment was to study the effect of simulated microgravity on the human body, such as vital signs (cardiac function, hormones, and muscle activity), body mass and body composition. During this study the Greek research team (under the leadership of Greek Aerospace Medical Association (GASMA) and the Laboratory of Medical Physics, Medical School of the Aristotle University of Thessaloniki) conducted an experiment to assess the effect of microgravity on sleep quality (Gkivogkli et al., 2016). The study employed PSG recordings 2 weeks before the bed rest initialization (Baseline Data Collection, BDC-14 phase), 21, 35, and 50 days after the experiment began (Head Down Tilt, HDT-21, 35, 50 phases) and finally 7 days after the end of the bed rest period (Recovery phase). Each of these recordings included an electroencephalogram (EEG) recorded with 19 Ag/AgCl electrodes positioned according to the International 10-20 System, as well as, other neurophysiological signals tracking the electrocardiographic (ECG), chin electromyographic (EMG) and electroocculographic (both vertical and horizontal EOG) activity. In this paper only the EEG recordings from the BDC-14 phase of the experiments were used for sleep stage classification purposes. This was done in order to avoid possible fluctuations in EEG recordings due to the experimental protocol. The sleep epochs from this experiment phase were manually staged by two experienced sleep experts as described in a later section.

### Pre-processing pipeline

**Figure 1:**
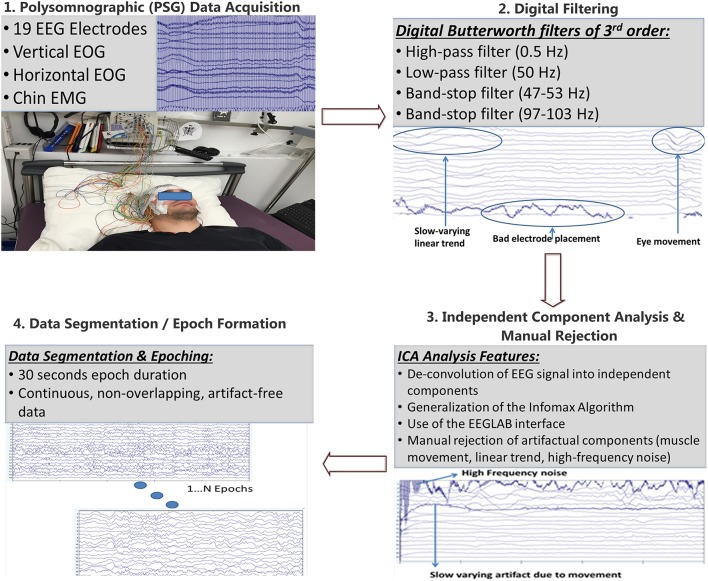
Pre-processing pipeline: After the EEG signal has been recorded it is segmented into five parts which are digitally filtered through Butterworth filters of 3rd order. Then, Independent Component Analysis is performed through the EEGLAB graphical interface and components corresponding to industrial noise, linear trends, muscle movements, bad electrode placement, eye blinks are visually rejected. Finally, epochs of 30 s of continuous, non-overlapping, artifact free data are formed according to the guidelines of the American Association of Sleep Medicine (AASM).

Prior to the synchronization analysis component, the pre-processing component of the pipeline was developed in order to filter the data and eliminate artifacts. The pre-processing component of the pipeline is presented in Figure 1. Since the polysomnographic data acquisition resulted in about 380 M samples, this volume was too large to be handled as a whole. So, the PSG data were divided in five equal segments for each subject. Then, the mean value $\bar{\text{x}}$ of the each channel **x** was computed as:

(1) $$\begin{array}{l} \bar{\text{x}}=\frac{1}{S}\sum_{i=1}^{S}{\text{x}}_{i} \end{array}$$

This mean value was subtracted from every sample point of the corresponding channel, resulting thus in a new signal, which has a mean value equal to zero rendering it suitable for computerized sleep staging since no DC bias is present. Then, digital filtering was performed through the Matlab environment. The filters used are third order Butterworth filters first described in 1930 by Stephen Butterworth (Bianchi and Sorrentino, 2007):

1. High pass filter with cut off frequency at 0.5 Hz (remove low frequency noise).
2. Low pass filter with cut off frequency at 50 Hz (remove high frequency noise).
3. Band stop filter in the range between 47 and 53 Hz (remove industrial noise).
4. Band stop filter in the range between 97 and 103 Hz (remove industrial noise harmonic).

The application of the abovementioned filters to the EEG signal results in the removal of the unwanted spectral content that is irrelevant to brain activity. This content originates from various sources, such as power line noise, interference from other devices, body/muscle movements, linear trends, bad electrode placement, ECG modulation and also various unknown sources that pollute the EEG data.

Then, the application of Independent Component Analysis, ICA took place (Bell and Sejnowski, 1995). ICA is a computational method capable of de-convolving a complex signal into independent components/sources that created the signal. This de-convolution is achieved through a generalization of Infomax, a method that aims to maximize information transfer by minimizing mutual information between input and output elements using a neural network, composed of neurons with a linear activation function. However this method is extended in the above paper in order to cover non-linear activation functions, sigmoid in this case, and with inputs that follow arbitrary distributions. The Infomax method de-convolves *N* inputs to *N* outputs that are as much as possible independent from one another. This is achieved through mutual information reduction, denoted as *I*, between the input and output, which is calculated as:

(2) $$\begin{array}{l} I\left(Y,X\right)\;=\;H\left(Y\right)-H\left(Y|X\right), \end{array}$$

where *Y* and *X* is the output and input of the neural network respectively and *H* denotes the entropy. This and the next analysis steps were performed through the EEGLAB graphic user interface (Delorme and Makeig, 2004).

From the independent components calculated during the previous step, only a subset is used to reconstruct the EEG signal. Specific components were manually rejected (pruned) as displayed in Figure 1. The number of components removed is not stable across segments, but usually ranged from 1-3 components. These components were considered to introduce noise and unwanted artifacts in the signal, which result from various noise sources such as eye movement, noise from the electrocardiogram, body movements as well as from other unknown sources. By rejecting these components a cleaner version of the signal can be reconstructed which is more suitable for further analysis. This process however tends to remove a portion of the EEG signal, and as such, while rejecting each component, the effect on the reconstructed signal was taken into account so that only but miniscule distortions are observed in the reconstructed EEG signal.

The final step of the pre-processing pipeline includes the partitioning of the EEG signal into 30 s epochs. This partitioning allows the calculation of the synchronization features and graph metrics for each epoch enabling the construction of a feature vector for each given epoch to be used for computer-assisted sleep staging. The proposed pipeline involves digital filtering adjusted to the 50 Hz power supply, which is valid in most European countries. However, it should be adjusted to the power supply standards of other countries such as the USA. Segmentation was also used to reduce the computational cost of the pipeline and is not an essential task. The same holds for the manual ICA component pruning, which may be omitted in order to allow fully automatic processing of EEG data used for sleep staging. The latter was hypothesized to improve the quality of derived features but may be not employed in real-time applications or in case of employing fewer electrodes. In our case ICA was used since an extended version of the same dataset would be used in forthcoming neuroimaging studies.

### Visual sleep scoring

To analyze collected data, visual sleep scoring was performed by adopting the criteria of American Academy of Sleep Medicine (AASM society) (AASM, 2007). Sleep data were divided into epochs of 30 s duration. During sleep stages alteration of brain rhythms was present since every sleep stage demonstrated different dominant rhythms. Descent to sleep (N1 stage) is the transition from wakefulness to the first sleep stage. The main characteristic of light sleep (N1), which lasts for 5% of total sleep time, is a low voltage mixed frequency (LVMF), pattern mainly consisted of alpha and theta activity. There are also vertex sharp waves. The N2 stage is the most common one (45% of the total sleep duration) and is characterized by sleep grapho-elements like K-complexes and spindles, while EEG slowing results in greater dominance of the theta rhythm. Deep sleep follows then and lasts ~ 20–25% of total sleep time with further EEG slowing resulting in delta rhythm and minimal eye and chin movement. Delta waves are maximally seen in frontal regions electrodes. Finally, the dreaming phase of sleep (REM stage) lasts 20–25% of sleep time. The EEG is consisted of alpha (1–2 Hz slower than wake Alpha) and theta waves. It is seen maximal over central regions. Body movement is minimal during that stage, while rapid eye movement and sawtooth waves co-exist with theta and alpha activity. There is also a noticeable increase of high frequency activity (beta and gamma). So, manual scoring was based on three electrodes located on the right hemisphere on frontal (F4), central (C4), and occipital (O2) regions. In case of severe noise contamination not corrected during the pre-processing pipeline the homologous electrode sites of the left hemisphere were used. The sleep experts also examined peripheral activity derived from EOG, ECG, and EMG signals as well as spectral analysis of the EEG channels. The sleep experts independently analyzed the same dataset. In case of disagreement for a given epoch, they discussed the issue and revisited the AASM manual until they reached a consensus. If the disagreement remained, this epoch was excluded from further analysis.

### Synchronization feature extraction

**Figure 2:**
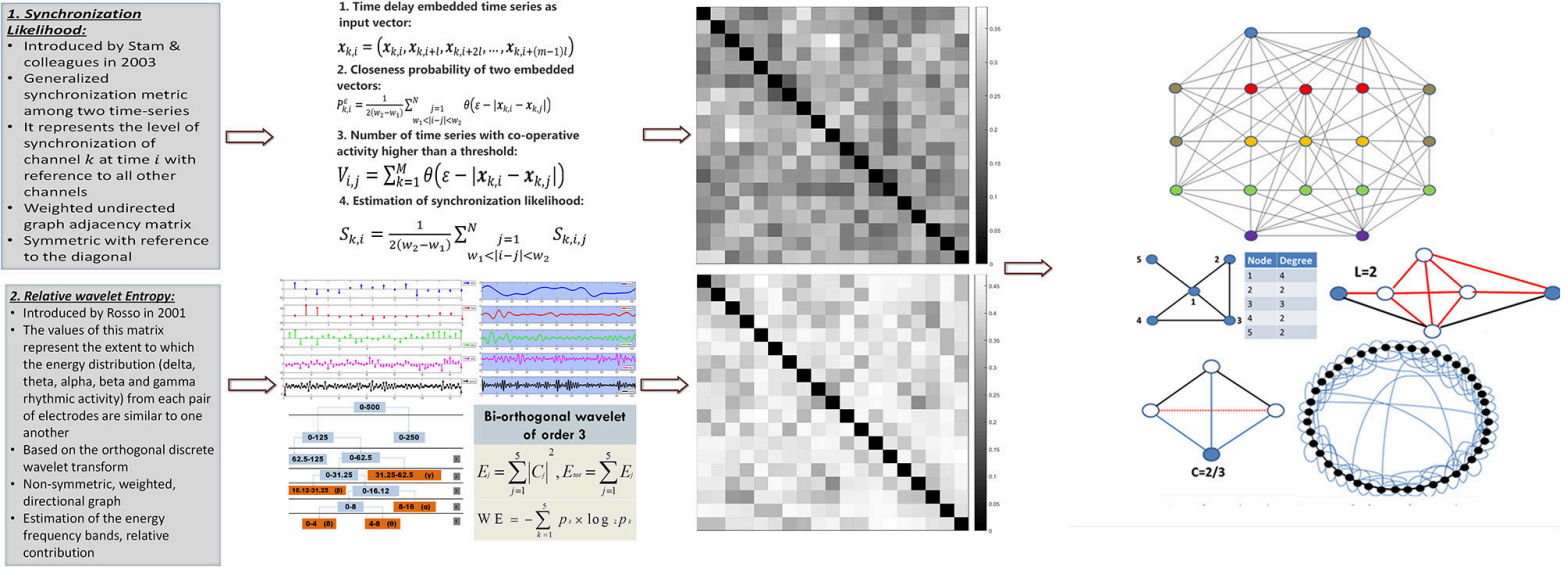
Feature extraction: For each epoch, the feature extraction procedure takes place during which features that quantify the macroscopic organization of the brain during sleep are extracted. Two alternative functional connectivity methodologies (Synchronization Likelihood and Relative Wavelet Entropy) are investigated. The synchronization matrices are used through concepts derived from graph theory (small-world property, cluster coefficient, characteristic path length, betweenness centrality, node degree) to form the feature vectors used for the computerized staging.

After the pre-processing steps the data are apt for the main processing phase, indicated as feature extraction component. As indicated in Figure 2, the feature extraction component employs alternatively two different methodologies for synchronization analysis. The first one calculates the synchronization likelihood between electrode pairs (Stam et al., 2003) and the second one calculates the relative wavelet entropy between the electrode pairs (Rosso et al., 2001). Each of these methods results in the calculation of a synchronization matrix which can be regarded as the adjacency matrix of a weighted graph. The nodes of the graph represent the electrode sites and the edges the interactions among electrode pairs. As such various graph metrics (small-world property, cluster coefficient, characteristic path length, etc.) are calculated for each given graph, following the calculation of the synchronization values.

#### Synchronization likelihood

Stam et al. (2003) defined synchronization likelihood as the measure of the degree of synchronization between two or more time series. This is based on the concept of generalized synchronization between two dynamic systems *Q*_1_ and *Q*_2_ and requires the existence of a continuous injective function *F* mapping the response of system *Q*_2_ to the responses of *Q*_1_according to *Q*_2_ = *F*(*Q*_1_). To calculate the synchronization likelihood of two system outputs, it is required to define the system states, signal values and a similarity metric between these values.

The general framework for the calculation of the synchronization likelihood matrices is presented in Stam and Van Dijk (2002) in which it assumed that there exist *M* simultaneously recorded signals, in this case the *k* channels recorded during the electroencephalogram, and assuming discrete time *i* = 1, …, *N*, where *N* denotes the number of samples. From each signal **x**_*k*_ the time-delay embedded vectors **x**_*k,i*_ are defined as:

(3) $$\begin{array}{l} {\text{x}}_{k,i}=\left({\text{x}}_{k,i},\;{\text{x}}_{k,i+l},{\text{x}}_{k,i+2l},\;\ldots ,\;{\text{x}}_{k,i+\left(m-1\right)l}\right), \end{array}$$

in which **x**_*k,i*_ corresponds to the values of signal **x** at channel *k* and time *i*, *l* denotes the time delay, and *m* the embedding dimension. The probability $P_{k,i}^{\varepsilon }$that two embedded vectors are closer to each other than a distance ε, for each channel *k* and time instance *i*, is equal:

(4) $$\begin{array}{l} P_{k,i}^{\varepsilon }=\frac{1}{2\left(w_{2}-w_{1}\right)}\sum_{\begin{array}{l} j=1\\ w_{1}\;<\;\left|i\;-\;j\right|<w_{2} \end{array}}^{N}\theta \left(\varepsilon -|{\text{x}}_{k,i}-{\text{x}}_{k,j}|\right) \end{array}$$

in which *w*_1_is the window for Theiler correction, *w*_2_the window for time resolution sharpening, θ is the Heaviside step function and |•| denotes the Euclidean distance. When $P_{k,i}^{\varepsilon_{k,1}}=p_{ref},\;p_{ref}\ll 1\;$the number of channels *V*_*i,j*_ within the current window that are closer than distance ε_*k,i*_ are:

(5) $$\begin{array}{l} V_{i,j}=\sum_{k=1}^{M}\theta \left(\varepsilon -|{\text{x}}_{k,i}-{\text{x}}_{k,j}|\right), \end{array}$$

which takes integer values in the range [0, *M*], representing the number of channels that are similar to each other.

The synchronization likelihood *S*_*k,i,j*_ for each channel *k* at times *i* and *j* is equal to:

(6) $$\begin{array}{l} S_{k,i,j}=\{\begin{array}{c} \frac{V_{i,j}-1}{M-1},\;if\left|x_{k,i}-x_{k,j}\right|<\varepsilon_{k,i}\\ 0,\;if\left|x_{k,i}-x_{k,j}\right|\geq \varepsilon_{k,i} \end{array} \end{array}$$

Finally, the synchronization likelihood *S*_*k,i*_ is averaged over *j* as:

(7) $$\begin{array}{l} S_{k,i}=\frac{1}{2\left(w_{2}-w_{1}\right)}\sum_{\begin{array}{l} j=1\\ w_{1}\;<\;\left|i\;-\;j\right|\;<\;w_{2} \end{array}}^{N}S_{k,i,j}. \end{array}$$

This synchronization likelihood metric represents the level of synchronization of channel *k* at time *i* with reference to all other channels. The synchronization likelihood values for the four sleep stages are stored in a *M* × *M* matrix **S** that is considered as a weighted undirected graph adjacency matrix, which is symmetric with reference to the diagonal.

#### Relative wavelet entropy

The second method of calculating a synchronization matrix is the Relative Wavelet Entropy (RWE) as defined in Rosso et al. (2001). The values of this matrix represent the extent to which the energy distribution (delta, theta, alpha, beta, and gamma rhythmic activity) from each pair of electrodes are similar to one another. Relative wavelet entropy is based on the orthogonal discrete wavelet transform (Simpson, 1993), which is an extension of the discrete wavelet transform, utilizing wavelets that define an orthogonal basis, used to decompose the initial signal. The continuous wavelet transform of a signal **x**(*t*) with scaling α ∈ ℝ^+*^ and at time *b*∈ ℝ is defined as:

(8) $$\begin{array}{l} X_{w}=\frac{1}{\sqrt{\alpha }}\int_{-\infty }^{+\infty }\text{x}\left(t\right)\bar{\psi }\left(\frac{t-b}{\alpha }\right)dt, \end{array}$$

where ψ(*t*) denotes a continuous function of time and frequency, known as mother wavelet, $\bar{•}$ is the conjugate complex number. Through function ψ it is possible to define a family of wavelet functions for different combinations of α and *b* as:

(9) $$\begin{array}{l} \psi_{a,b}\left(t\right)=|\alpha {|}^{-\frac{1}{2}}\psi \left(\frac{t-b}{\alpha }\right). \end{array}$$

Since an orthogonal basis is required this family must define an orthogonal basis in Hilbert space *L*^2^(ℝ), abiding to two conditions, the first one being $\alpha_{j}=2^{-j}$ and the second one $b_{j,k}=2^{-j}k$ with *j, k*∈*Z* and is defined as:

(10) $$\begin{array}{l} \psi_{j,k}\left(t\right)=2^{\frac{j}{2}}\psi \left(2^{j}t-k\right). \end{array}$$

For a given uniformly sampled signal **x** which is decomposed in *N* levels the orthogonal wavelet transform is given by:

(11) $$\begin{array}{l} X_{t}=\sum_{j=-N}^{-1}\sum_{k}C_{j}\left(k\right)\psi_{j,k}\left(t\right) \end{array}$$

in which *C*_*j*_(*k*) denotes the wavelet coefficients at times *k*. The amplitude of these coefficients quantifies the degree of similarity between the mother wavelet and the signal, while its sign describes if this similarity has positive or negative polarity. Through the calculated wavelet coefficients it is possible to calculate the overall energy as:

(12) $$\begin{array}{l} E_{tot}=\sum_{j<0}\sum_{k}{\left|C_{j}\left(k\right)\right|}^{2}. \end{array}$$

The relative energies *p*_*j*_ for each level are given by the ratio of the energy at level *j* over the total energy. Finally the relative entropy between two signals *p* and *q* at level *j* is given through the Shannon entropy (Segal, 1960) as:

(13) $$\begin{array}{l} H\left(p|q\right)=-\sum_{j<0}p_{j}\ln\left(\frac{p_{j}}{q_{j}}\right), \end{array}$$

which calculated for each pair of electrodes defines a synchronization matrix similar to the one calculated through synchronization likelihood. However the main difference is that relative wavelet entropy represents the adjacency matrix of a weighted directional graph which is not symmetric.

Apart from the relative wavelet entropy values five more features are calculated through the above process. These features are the ratios of the energies of the five main brain wave rhythms, namely delta (δ), theta (θ), alpha (α), beta (β), and gamma (γ) over the total energy of the signal. These features are calculated during the four different sleep stages, since different rhythms are prominent in the EEG recordings and thus it is possible to further differentiate between the sleep stages through the above energy ratios.

#### Graph metrics

Having calculated the synchronization matrices, and regarding them as adjacency matrices, it is possible to calculate various graph metrics that describe the connectivity between the electrodes and therefore the interactions between different neuronal groups. The metrics (Deuker et al., 2009; Nicosia et al., 2013; Frantzidis et al., 2014b; Deo, 2017) used in this paper are presented in Table 1. These metrics range from the fundamental network properties (node degree and network density) to global network characteristics quantifying the overall network performance (small-world property), the network's information flow across the network nodes (characteristic path length) and the capacity of local information processing as expressed by the mean cluster coefficient. Centrality metrics as the relative betweenness centrality have been used to quantify the importance of each specific node to the integration of the information capacity. These features have been visualized in Figure 2. More information regarding these network metrics may be found in (Frantzidis et al., 2014b).

**Table 1:** Graph metrics.

**Network Metric**	**Network Description**
Node degree	Number of immediate neighbors of each node
Clustering coefficient	Strength of node connection
Characteristic path efficiency and length	Length and efficiency of the shortest path connecting all nodes
Connection density	Number of connections present in a graph divided by the total possible connections of the same graph
Centrality	Number of shortest paths connecting all other pairs of nodes that incorporate the given node
Small world metric	Quantifies the average distance between nodes in a graph

### Classification methodology component

This is the core component of the proposed pipeline, since it employs the features extracted from the synchronization and brain network analysis in order to perform the computerized sleep staging based on functional, system-level information. After calculating the synchronization metrics and graph metrics it is possible to construct a feature vector for each epoch, to be used for semi-automatic sleep stage classification. Both synchronization matrices have dimensions 19 × 19and must be vectorized in order to allow classifier training. However the main diagonal contains the synchronization values of each electrode with itself, information that is not needed, as each electrode is synchronous to itself. Therefore the values of the diagonal are omitted. The matrix calculated through synchronization likelihood is symmetric, containing redundant information as each synchronization value is observed twice. As such only the strictly lower triangular matrix is vectorized resulting in 171 distinct synchronization values, which along with the 7 graph metrics calculated results in a feature vector of 178 dimensions. In the case of relative wavelet entropy the synchronization matrix is not symmetric and all synchronization values are incorporated in the feature vector, except the values of the main diagonal. This results in 342 distinct values along with 5 graph metrics and 5 brain rhythm energy ratios resulting in a final feature vector of 352 dimensions. All values were normalized per feature in the range [0, 1].

From the initial set of data, 2,447 epochs were manually selected after the pre-processing step, in which noise did not distort the signal significantly. These epochs were randomly divided in two sets, one training and one testing each containing 1,711 (70%) and 736 (30%) epochs respectively. The randomization was performed on the entire dataset and not per participant. Moreover, the number of epochs per participant was not equal, since some of them had no N1 epochs and some other did not successfully transition to deep sleep (N3 & REM). Each set contained equal proportions of each sleep stage. The training and testing sets are subsequently used to train and evaluate the accuracy of three different classifiers, namely k-Nearest Neighbors classifier (k-NN), Support Vector Machines (SVM) (Chang and Lin, 2011) and Neural Networks (NN). It must be noted that due to the significant differences that are observed in the duration of each sleep stage the classification task is quite difficult.

For each classifier used in the experimental procedure a set of different parameter values where used in order to assess the sleep staging potential of each one. In more detail, in the case of the k-Nearest Neighbor classifier experiments were ran by adjusting the number of nearest neighbors, for *k* = 1, 3, and 5, and three distance metrics namely the Euclidean and Cityblock distances and Cosine similarity. For SVM's, the parameters studied were the value of the penalty imposed on misclassified training data, *C* = 0.1, 10, 100, and the kernel type along with each kernel's parameters. Three different kernel types where used the linear, polynomial and the Gaussian kernel. Finally experiments where ran on neural networks with one and two hidden layers of various sizes. The SVM and NN parameters were optimized using 10 fold cross-validation.

## Results

**Figure 3:**
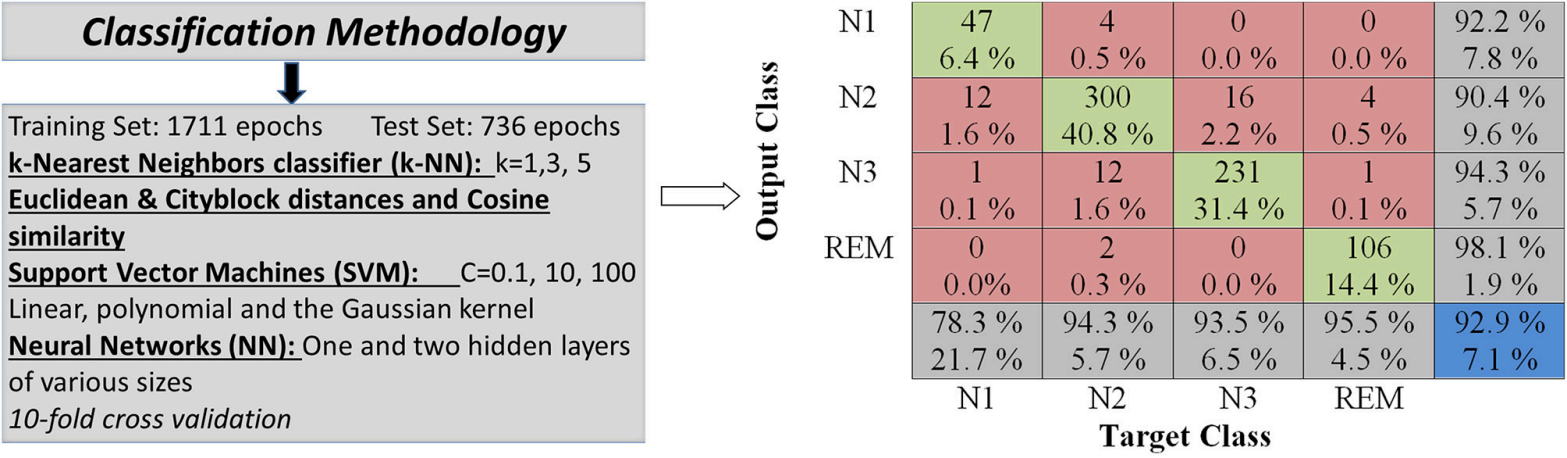
Computer-assisted classification is performed with various classifiers such as the k-Nearest Neighbors (kNN), Support Vector Machines (SVMs) and Neural Networks (NNs). Various classification parameters are employed in a comparative analysis.

The experimental results are summarily presented in Table 2. In this table the classification accuracies are displayed for both training and test sets as well as for each feature extraction method, classifier and classifier parameter set, an outline of which is displayed in Figure 3.

**Table 2:** Sleep stage classification results.

**Classifier Parameters**		**Classification Accuracy**			
**k-Nearest Neighbors**		**Synchronization Likelihood**		**Relative Wavelet Entropy**	
**k**	**Distance**	**Train (%)**	**Test (%)**	**Train (%)**	**Test (%)**
1	Euclidean	100.00	72.55	100.00	89.95
3	Euclidean	85.80	74.86	96.26	90.90
5	Euclidean	83.40	76.22	94.39	91.44
1	Cityblock	100.00	67.93	100.00	88.18
3	Cityblock	84.40	71.33	95.79	89.40
5	Cityblock	80.48	73.10	94.16	89.95
1	Cosine	100.00	73.91	100.00	89.95
3	Cosine	88.66	77.17	96.26	91.85
5	Cosine	85.97	78.26	94.45	91.44
**Support Vector Machines**		**Synchronization Likelihood**		**Relative Wavelet Entropy**	
**C**	**Kernel**	**Train (%)**	**Test (%)**	**Train (%)**	**Test (%)**
0.1	Linear	73.35	71.74	87.61	85.05
10	Linear	89.42	79.35	99.24	90.22
100	Linear	93.28	76.49	100.00	90.49
0.1	Polynomial, *d* = 3	53.83	52.72	99.65	91.44
10	Polynomial, *d* = 3	90.77	80.98	100.00	91.58
100	Polynomial, *d* = 3	99.59	82.07	100.00	91.58
0.1	Polynomial, *d* = 5	51.14	51.22	100.00	91.58
10	Polynomial, *d* = 5	86.44	78.94	100.00	91.58
100	Polynomial, *d* = 5	98.48	82.34	100.00	91.58
0.1	Gaussian, σ_SL_ = 1.95 σ_RWE_ = 0.25	71.65	67.53	80.30	78.13
10	Gaussian, σ_SL_ = 1.95 σ_RWE_ = 0.25	100.00	**86.82**	100.00	**92.93**
100	Gaussian, σ_SL_ = 1.95 σ_RWE_ = 0.25	100.00	86.82	100.00	92.80
0.1	Gaussian, σ_SL_ = 1.45 σ_RWE_ = 0.75	72.71	69.57	78.73	74.73
10	Gaussian, σ_SL_ = 1.45 σ_RWE_ = 0.75	100.00	86.28	100.00	92.66
100	Gaussian, σ_SL_ = 1.45 σ_RWE_ = 0.75	100.00	86.28	100.00	92.66
**Neural Networks**		**Synchronization Likelihood**		**Relative Wavelet Entropy**	
**Layer 1**	**Layer 2**	**Train (%)**	**Test (%)**	**Train (%)**	**Test (%)**
10	–	85.80	78.26	94.16	87.77
30	–	83.82	79.48	96.67	90.49
50	–	84.04	78.94	95.79	90.22
100	–	86.62	80.16	94.39	88.59
50	50	86.73	78.13	96.20	89.54
100	50	86.09	79.89	97.49	89.13
100	100	86.97	79.08	95.27	88.72

*Bold values are the maximum achieved accuracy for each feature extraction method*.

### Synchronization likelihood results

The first classifier evaluated using the feature vectors calculated through synchronization likelihood is the k-NN classifier. For *k* = 1 the accuracy on the training set is, as expected, equal to 100% for all distance metrics used. This is true since all training samples are closest to themselves, at zero distance and with similarity equal to one, and as such are always classified correctly and therefore these values are omitted. Using the Euclidean distance as the distance metric leads to a maximum accuracy equal to 85.80% for the training data and 76.22% for the test data for *k* = 3 and *k* = 5 respectively. For the Cityblock distance the training set accuracy is lower than the Euclidean distance equal to 84.40 and 73.91% for the training set, for the same number of nearest neighbors mentioned above. The best results with the k-NN classifier are given through the cosine similarity metric reaching an accuracy of 88.66% for the training data (*k* = 3) and 78.26% for the test set (*k* = 5).

Three different kernels where evaluated using SVM classifiers. The first one is the linear kernel that provided a maximum accuracy percentage equal to 93.28% for the training data and *C* = 100 and 79.35% for the test data with *C* = 10. The polynomial kernel was used with two different degrees, the first one *d* = 3, which reached 99.59% (*C* = 100) in the training set and 82.07% for the test set with the same value of C. For *d* = 5 the maximum accuracy was 98.48 and 82.34% with *C* = 100 for the training and test data respectively. The Gaussian kernel was also evaluated, achieving the maximum accuracy for the test data equal to 86.82% with σSL = 1.95 and *C* = 10. This kernel achieved perfect accuracy for the training data for various parameter values, hinting that the classifiers may be overfitted but still capable of performing well on the test data.

In Table 3 the confusion matrix for the Gaussian kernel SVM with σ = 1.95 which achieved the highest accuracy rate is presented. As expected the epochs belonging to the N1 sleep stage are the most difficult to differentiate from the other stages. This is evident from the fact that from the total of 60 N1 epochs in the test set only 39 are classified correctly, an accuracy equal to 65%. In the case of the N2 and N3 sleep stages accuracy rates are higher reaching 89.9 and 85.8% respectively. The highest accuracy is achieved for the REM stage equal to 91.9%.

**Table 3:** Confusion matrix for the Gaussian kernel SVM classifier (σ = 1.95) that achieved maximum accuracy with synchronization likelihood features.

Output Class	N1	39 5.3%	4 0.5%	1 0.1%	0 0.1%	88.6% 11.4%
	N2	18 2.4%	286 38.9%	31 4.2%	6 0.8%	83.9% 16.1%
	N3	3 0.4%	23 3.1%	212 28.8%	3 0.4%	88.0% 12.0%
	REM	0 0.0%	5 0.7%	3 0.4%	102 13.9%	92.7% 7.3%
		65.0% 35.0%	89.9% 10.1%	85.8% 14.2%	91.9% 8.1%	86.8% 13.2%
		N1	N2	N3	REM	
Target Class						

Neural networks of various sizes were the third type of classifier used in the experimental process. For networks with one hidden layer the highest accuracy percentage equal to 86.62% for the training data was achieved with a hidden layer size equal to 100 which also provided the highest accuracy of 80.16% for the test data. Larger networks with two hidden layers failed to provide higher accuracies for the test data, the highest being 79.89% for layers of size 100 and 50, but provided a maximum of 86.97% for the training data. This observation is likely due to the overfitting of the neural networks since large networks require a large number of data samples to avoid overfitting and achieve adequate generalization.

### Relative wavelet entropy (RWE) results

Similar experiments were ran for the feature vectors constructed through Relative Wavelet Entropy (RWE), although the accuracy percentages were generally higher compared to the one mentioned above. For the k-NN classifier the classification results for the training data and *k* = 1, are omitted for the reasons mentioned in the previous section. With the Euclidean distance the highest classification accuracy attained is 96.26% (*k* = 3) and 91.44% (*k* = 5) for the training and test sets respectively. The Cityblock metric fails to achieve higher accuracy rates compared to the Euclidean distance regardless of data set and values of k. The highest accuracy of the k-NN classifier on the test set is achieved with the cosine similarity metric with *k* = 3 equal to 91.85%.

Support Vector Machines along with RWE features achieved the maximum accuracy percentage reported in this paper reaching 92.93% on the test data with a Gaussian kernel with σ_RWE_ = 0.25 and *C* = 10. The other kernels also achieved high accuracy rates with the linear kernel achieving a maximum of 90.49% for *C* = 100, and the polynomial kernel reaching 91.58% for various parameter values. Perfect accuracy is reported through SVM's for various kernels and parameter values.

The confusion matrix for the Gaussian kernel SVM with σ = 0.25 is presented in Table 4. This classifier achieved the highest accuracy rate for the RWE features. Similarly to the results mentioned on the previous section, the lowest accuracy is reported for the N1 sleep stage equal to 78.3%. The accuracy rates for the N2 and N3 sleep stages are higher equal to 94.3% and 93.5% respectively. Again the highest accuracy is achieved for the REM stage equal to 95.5%.

**Table 4:** Confusion matrix for the Gaussian kernel SVM classifier (σ = 0.25) that achieved maximum accuracy with relative wavelet entropy features.

Output Class	N1	47 6.4%	4 0.5%	0 0.0%	0 0.0%	92.2% 7.8%
	N2	12 1.6%	300 40.8%	16 2.2%	4 0.5%	90.4% 9.6%
	N3	1 0.1%	12 1.6%	231 31.4%	1 0.1%	94.3% 5.7%
	REM	0 0.0%	2 0.3%	0 0.0%	106 14.4%	98.1% 1.9%
		78.3% 21.7%	94.3% 5.7%	93.5% 6.5%	95.5% 4.5%	92.9% 7.1%
		N1	N2	N3	REM	
Target Class						

Neural networks failed to surpass the sleep staging accuracy of SVM's with one hidden layer networks achieving 96.67% accuracy on the training data and 90.49% for test set with a layer of size 30. For networks with two hidden layers the highest accuracy for the training set was 97.49% for layers of size 100 and 50, and a maximum accuracy for the test set equal to 89.54% for two layers of size 50.

## Discussion

In this paper two different methods of functional connectivity estimation (Synchronization Likelihood / SL and Relative Wavelet Entropy/RWE) have been utilized in computerized sleep staging. Results indicate that functional connectivity features, derived either from Synchronization Likelihood or Relative Wavelet Entropy along with the associated graph metrics computed and extracted from sleep epochs are suitable for sleep stage classification purposes. High sleep staging accuracies on the test data reported above support this evidence building, as accuracies from 86.82 to 92.93% were obtained for SL and RWE respectively, either one provided by a Support Vector Machine with a Gaussian kernel. Obviously, RWE features seems to be preferable as it achieves better accuracy attained than that achieved by synchronization likelihood features. The system's accuracy is among the highest ones reported by the literature (Bajaj and Pachori, 2013; Lajnef et al., 2015). So, it is regarded as adequate for assisting the role of sleep experts during manual staging. Moreover, we should note that visual sleep staging is highly dependent on the expert as presented in Danker-hopfe et al. (2009).

The two feature extraction methods used in this paper, synchronization likelihood and relative wavelet entropy, quantify the interaction between each pair of electrodes serving as a functional connectivity metric. This is contrary to the most state-of-the-art methods that mainly utilize specific spectral, statistical and time domain features. Furthermore, using metrics derived from graph theory it is possible to describe electroencephalographic activity during sleep on a higher level, providing a macroscopic and holistic system view. Beyond the above features, the energy ratios of the five main brain wave rhythms were also calculated. These features provide an integral and functional description of electrode activity during sleep as it is possible to estimate the overall energy distribution and interaction between electrode pairs. Also, in the experimental process, the unequal duration of each sleep stage was taken into account, and no attempt was made to have the same number of epochs for each sleep stage. This definitely rendered the classification of specific sleep stages problematic, at the same time adhered to the realistic scenario in which sleep epochs are not uniformly distributed over the sleep stages. Finally, the proposed semi-automatic sleep staging methods rely on time domain and spectral features while there is limited research on the macroscopic organization of the brain and the study of the brain as a network. Both of these concepts are pursued in this paper. Apart from studying/quantifying the interactions between specific regions, and how these change during the various sleep stages, features extracted through graph theory and the energy ratios provide an integrated and holistic view of brain functionality during sleep, which can also be used for further analysis of the brain network.

Classification results are summarized in the confusion matrices presented in Tables 3, 4, where red squares denote errors, green squares correct classification, gray squares classification percentages for each sleep stage and the blue square represents the average accuracy overall. As it can be observed from the Tables 3, 4, the N1 sleep stage is the hardest to differentiate from the others probably due to the proportionately small number of available epochs for training. While accuracy rates for the N2 and N3 stages are higher than the N1 stage, it is clear that these sleep stages are not easily distinguished from one another due to the common characteristics they share. It is also observed that N2 is distinguished easier than N3, 89.9% over 85.8% a significant difference for synchronization likelihood features and 94.3% over 93.5% for RWE features. The highest accuracy rate in both feature extraction methods studied is achieved for the REM sleep stage. This is likely due to REM's unique characteristics making its classification easier.

The proposed methodologies are capable of achieving high accuracy rates but there is room for further development, for instance achieving an accuracy of over 95% and experimenting with a larger dataset. Furthermore, the effectiveness of the proposed methods should also be tested with different electroencephalogram recording setups with fewer or more electrodes and varying levels of noise.

As mentioned above SVMs with a Gaussian kernel achieved the highest classification accuracies. However this does not indicate that this classifier is the most suitable for sleep stage classification. For a different dataset, different classifier values or perhaps a different classifier altogether may provide higher classification accuracies. Therefore, there is need for further investigation of the applicability and robustness of the aforementioned classifiers on different dataset gathered from different devices and from heterogeneous populations (e.g., participants with different ages and patients suffering from sleep disorders). The inclusion of functional connectivity features, which track dynamic interactions during sleep, is hypothesized to facilitate the generalization of the proposed framework. Another interesting observation on the classifiers evaluated in this paper is that in some cases SVMs provide higher accuracies than neural networks. This is likely due to the number of available data, since neural networks require very large datasets to avoid overtraining and achieve sufficient generalization. This is probably also the reason why the k-NN classifier surpasses the accuracy of neural networks on the test set.

Future work in automatic sleep stage classification will be targeted at experimenting with a larger number of data samples and extracting and selecting finer features. Employing more epochs in our subsequent work will allow us to apply deep learning methodologies through convolutional neural networks and restricted Boltzmann machines. We will also investigate the feasibility of using cortical activations instead of sensor-level data. This may result in better classifications results. Another approach to increase sleep staging accuracy would be to utilize not only electroencephalographic recordings but to also incorporate features from other neurophysiological signals recorded during sleep such as features extracted from electrocardiographic, electrooculographic and electromyographic activity. Furthermore, we should highlight that current analysis was focused on the interactions among EEG activity on sensor level. However, this type of analysis faces several limitations due to the low spatial resolution of the recording modality, which results in volume conduction artifacts due to linear mixing of sources into sensors (Haufe et al., 2013; Frantzidis et al., 2014a). Future expansion of our work would focus on estimating cortical functional connectivity metrics, which may enhance the robustness of our system. Another limitation of our study is that the randomization procedure may assign data from the same participant to both the training and testing sets. However, we should note that the pool of participants is relatively small (23 participants in total). Moreover, our study was based on a head-down tilt (6°) bed-rest study, aiming to assess pathological deviations from normal sleep due to the microgravity effect. So, we are interested on identifying features which facilitate the sleep quality quantification and body-system interactions. This was the reason that motivated us to perform the specific epoch assignment to training and test groups and would be difficult with the current dataset to distribute whole participants into either the training or the testing set. We acknowledge that this may provide optimistic results based on individual patterns persistent across epochs of the same sleep stage. In the future our dataset will incorporate sleep epochs from both normal and pathological sleep and will present a greater challenge in achieving high accuracy sleep staging.

Our system's accuracy rate can potentially aid sleep experts in manually scoring sleep epochs, reducing the required time cost and possible errors, inherent to visual sleep staging. However our approach should be further validated by different data sets derived from both healthy and pathological populations such as those found in the SIESTA Project (Klosh et al., 2001) and the Sleep-EDF database (Kemp et al., 2000). The interaction with the above and other databases could be achieved through online applications that allow data processing and sharing such as the platform presented in Frantzidis et al. (2010) and Beier et al. (2017). Another project our system could take part is the SmokeFreeBrain project which records PSG activity before and after varenicline-based (pharmacological) smoking cessation intervention (http://smokefreebrain.eu/). These heterogeneous sleep data are expected to further improve the methodology applicability offering a publicly available web-based sleep analyzer aiming to enhance international and inter-disciplinary cooperation in the field of automatic sleep research.

Concluding, the proposed methodologies capable of providing almost optimal sleep stage classification, with accuracy above 90% compared to expert staging, through functional connectivity feature extraction and metrics derived from graph theory.

## Author contributions

PC developed the scripts, analyzed the data and prepared the initial draft of the manuscript; CF collected the data, guided the analysis and revised the manuscript; PG assisted the data collection, performed manual sleep staging and guided the feature selection procedure; PB conceived the analysis pipeline, guided the study and revised the manuscript; CK-P conceived the concept of the study, co-guided the study and revised the manuscript.

### Conflict of interest statement

The authors declare that the research was conducted in the absence of any commercial or financial relationships that could be construed as a potential conflict of interest.
